# *Salmonella* inhibits tumor angiogenesis by downregulation of vascular endothelial growth factor

**DOI:** 10.18632/oncotarget.7038

**Published:** 2016-01-27

**Authors:** Dom-Gene Tu, Wen-Wei Chang, Song-Tao Lin, Chun-Yu Kuo, Yu-Tzu Tsao, Che-Hsin Lee

**Affiliations:** ^1^ Department of Nuclear Medicine, Ditmanson Medical Foundation, Chia-Yi Christian Hospital, Chia-Yi, Taiwan; ^2^ Department of Food Science and Technology, Chia Nan University of Pharmacy and Science, Tainan, Taiwan; ^3^ College of Health Sciences, Chang Jung Christian University, Tainan, Taiwan; ^4^ Department of Biomedical Sciences, College of Medical Science and Technology, Chung Shan Medical University, Taichung, Taiwan; ^5^ Department of Medical Research, Chung Shan Medical University Hospital, Taichung, Taiwan; ^6^ Department of Microbiology, School of Medicine, China Medical University, Taichung, Taiwan; ^7^ Graduate Institute of Basic Medical Science, School of Medicine, China Medical University, Taichung, Taiwan; ^8^ Department of Medicine, Taoyuan General Hospital, Taoyuan, Taiwan; ^9^ Department of Biological Sciences, National Sun Yat-sen University, Kaohsiung, Taiwan

**Keywords:** Salmonella, tumor-targeting, angiogenesis, hypoxia-inducible factor-1α, vascular endothelial growth factor

## Abstract

*Salmonella* is a Gram-negative, facultative anaerobe that is a common cause of host intestinal infections. *Salmonella* grows under aerobic and anaerobic conditions, and it has been proven capable of inhibiting tumor growth. However, the molecular mechanism by which *Salmonella* inhibits tumor growth is still unclear. Angiogenesis plays an important role in the development and progression of tumors. We investigated the antitumor effect of *Salmonella* in a syngeneic murine tumor model. Hypoxia-inducible factor-1 (HIF-1)α plays a significant role in tumor angiogenesis. We examined the molecular mechanism by which *Salmonella* regulated vascular endothelial growth factor (VEGF), which is an important angiogenic factor. The expression of VEGF in tumor cells was decreased by treatment with *Salmonella*. The conditioned medium from *Salmonella*-treated cells inhibited the proliferation of endothelial cells. *Salmonella* inhibited the expression of HIF-1α as well as downregulated its upstream signal mediator protein kinase B (AKT). *Salmonella* significantly inhibited tumor growth *in vivo*, and immunohistochemical studies of the tumors revealed decreased intratumoral microvessel density. These results suggest that *Salmonella* therapy, which exerts anti-angiogenic activities, represents a promising strategy for the treatment of tumors.

## INTRODUCTION

Attenuated *Salmonella* has been demonstrated to inhibit tumor growth in a broad range of human and mouse tumors [1–9]. There are many advantages of using *Salmonella* for cancer treatment, including tumor-targeting, immunostimulation and low cost [10, 11]. *Salmonella* stimulates host immunity and reduces tumor growth [12, 13]. Additionally, *Salmonella* was shown to replicate much more in tumors than in normal tissue and to target to the hypoxic regions in tumors [14, 15].

A hypoxic microenvironment is a hallmark of many solid tumors. Hypoxia is also associated with a more malignant phenotype, affecting genomic stability, apoptosis, autophagy, angiogenesis and metastasis [16]. Induction of angiogenesis plays an important role in the development and progression of most tumors. Targeting angiogenesis to inhibit tumor growth is one of the promising therapeutic approaches for tumor treatment [17]. Interestingly, a previous study revealed that mice treated with *Salmonella* alone, compared with those treated with Phosphate-buffered saline (PBS), showed slightly reduced intratumoral microvessel density [15]. To date, a possible interaction of *Salmonella* with tumor cells has not been examined. Herein, we propose a role for *Salmonella* in controlling tumor growth by reducing vascular endothelial growth factor (VEGF) expression.

## RESULTS

### *Salmonella* reduced the proliferation of endothelial cells

**Figure 1:**
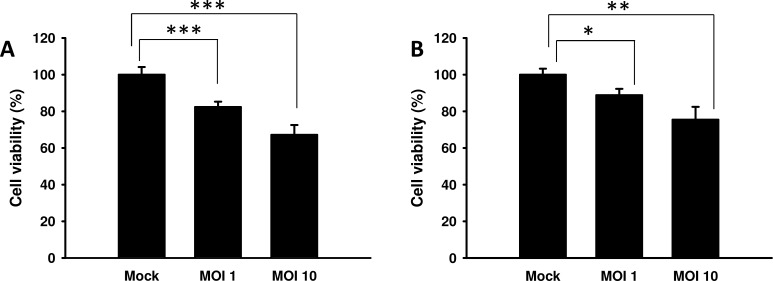
The effect of medium conditioned with *Salmonella* (S.C.) - treated tumor cells on endothelial cells The conditioned medium of *Salmonella*-treated B16F10 **A.** and 4T1 **B.** cells reduced the proliferation of endothelial cells. The HEMC-1 cells treated with the conditioned medium of *Salmonella*-treated tumor cells were examined *via* proliferation activity. Cell viability was assessed by the WST-1 assay. Data shown are the mean ± SD (*n* = 4). Multiplicity of infection (MOI). * *p* < 0.05; ** *p* < 0.01; *** *p* < 0.001.

Previous studies found that the conditioned medium from *Salmonella*-infected tumor cells moderately inhibited the proliferation of endothelial cells [14]. Herein, the proliferation of endothelial cells was measured in the conditioned medium of tumor cells treated with *Salmonella*. The results show that the amounts of endothelial cells were lowest when endothelial cells were cultured with the conditioned medium of B16F10 cells treated with the highest dose of *Salmonella* (multiplicity of infection (MOI) = 10) compared with the control group (Figure 1A). Similar results were observed in 4T1 cells treated with *Salmonella* (Figure 1B). Collectively, these results suggest that *Salmonella* may decrease the proliferation of endothelial cells through reducing growth factors produced by tumor cells. The above findings prompted us to further explore the detailed mechanism underlying the anti-angiogenic effects of *Salmonella* in tumors.

### *Salmonella* reduced HIF-1α expression through the phospho-protein kinase B (P-AKT)/ phospho-mammalian target of the rapamycin (P-mTOR) pathway

**Figure 2:**
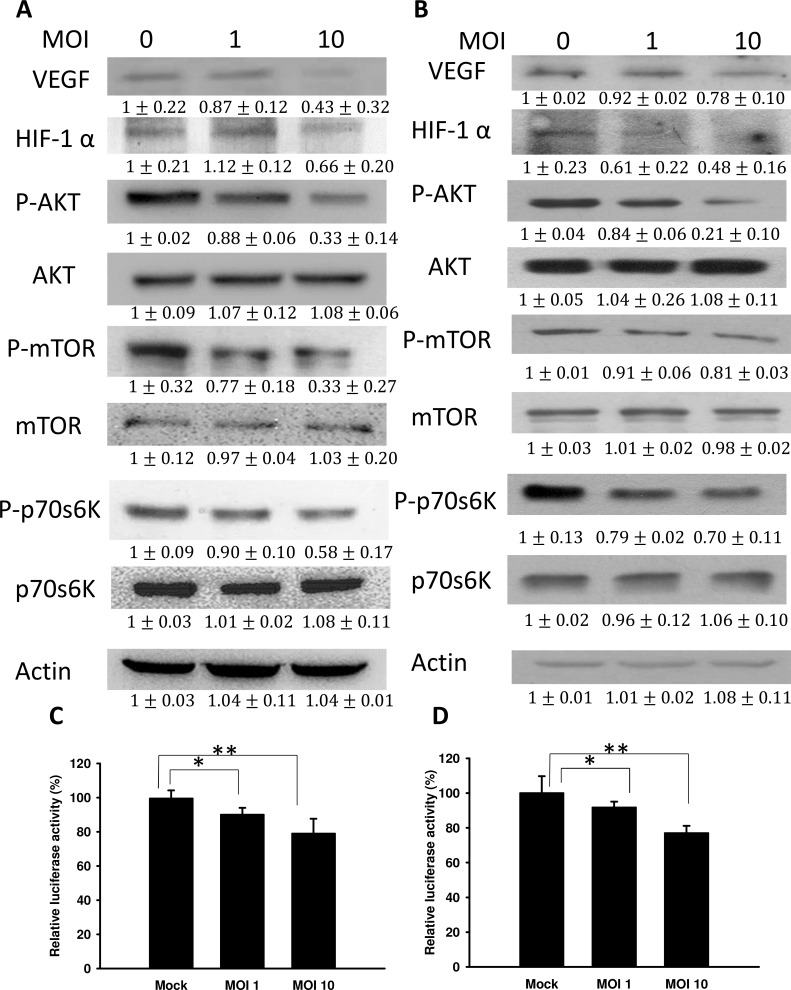
*Salmonella* (S.C.) regulated the expression of HIF-1α, vascular endothelial growth factor (VEGF), and AKT/mTOR signaling pathway components The expression of VEGF, HIF-1α, AKT, mTOR, and p70s6K was measured in B16F10 **A.** and 4T1 **B.** cells by Western blot analysis. β-actin expression served as a loading control and as an indicator of total protein levels. Inserted values indicate relative protein expression in comparison with β-actin (MOI=0). Each experiment was repeated three times with similar results. The data shown are the mean ± SD (*n* = 3). *Salmonella*-treated B16F10 **C.** and 4T1 **D.** cells were cotransfected with pCLNCX-6× HRELuc and pTCYLacZ plasmids. At 6 h post-transfection, their luciferase activities were determined and normalized with β-gal activity. Multiplicity of infection (MOI). Data shown are the mean ± SD (*n* = 4). * *p* < 0.05; ** *p* < 0.01.

Vascular endothelial growth factor (VEGF), a major growth factor, can induce angiogenesis during tumor growth. As shown in Figure 2A and 2B, the protein levels of VEGF were dramatically decreased in *Salmonella*-treated tumor cells. Because HIF-1α can induce VEGF expression, the expression of HIF-1α in tumors was measured. The expression of HIF-1α was decreased in *Salmonella*-treated tumor cells (B16F10 and 4T1). The extent of hypoxia responsiveness of the hypoxia-response element (HRE) reporter assay in two tumor cell lines varied, ranging from 0.91-0.76-fold compared with control cells (Figure 2C and 2D). The levels of hypoxia responsiveness were lower in *Salmonella*-treated tumor cells (B16F10 and 4T1) than those in mock-infected cells. Because *Salmonella* can influence HIF-1α protein expression and thereby abrogate HIF-1α-mediated transcriptional activity in tumor cells, we wanted to identify the signaling pathway affected by *Salmonella* during tumor angiogenesis. Some studies demonstrated that the protein kinase B (AKT)/ mammalian target of rapamycin (mTOR) signaling pathway can promote HIF-1αprotein synthesis through phosphorylation of protein translational regulators, such as ribosomal p70S6 kinase (p70S6K1) [18, 19]. As expected, the elevated phosphorylation of AKT, mTOR, and p70S6K in B16F10 and 4T1 cells was significantly diminished by *Salmonella* treatment (Figure 2A and 2B). Taken together, treatment with *Salmonella* decreased the phosphorylation of AKT, mTOR and p70S6K in a dose-dependent manner, indicating downregulation of the AKT/mTOR/p70S6K/HIF-1α/VEGF pathway in tumor cells treated with *Salmonella*. These results indicated that reduction of VEGF expression by *Salmonella* in tumor cells was associated with inhibition of the AKT/mTOR/p70S6K pathway.

### *Salmonella* reduced VEGF *via* inhibiting the AKT signaling pathway

**Figure 3:**
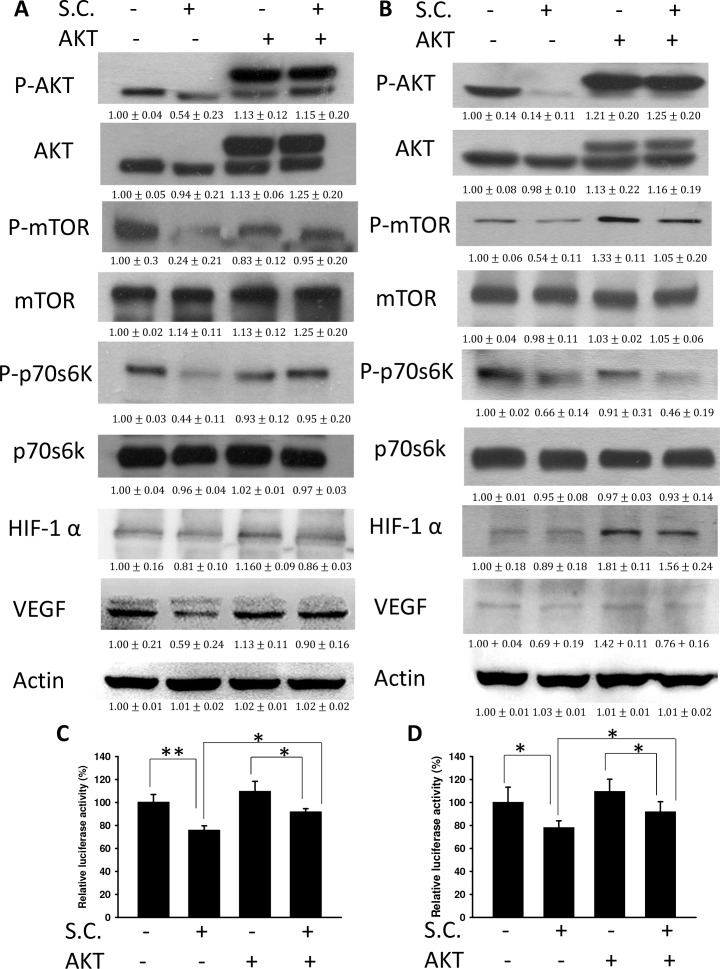
Constitutively active AKT reduced the effects of Salmonella (S.C.) The B16F10 and 4T1 cells were transfected with a constitutively active AKT plasmid (5 μg) at 37°C for 16 hours prior to infection with *Salmonella* (MOI = 10) for 4 h. The expression of VEGF, HIF-1α, AKT, mTOR, p70s6K protein in B16F10 **A.** and 4T1 cells **B.** was determined. β-actin expression served as a loading control and as an indicator of total protein levels. Inserted values indicate relative protein expression in comparison with β-actin (MOI=0). Each experiment was repeated three times with similar results. Data shown are the mean ± SD (*n* = 3). *Salmonella*-treated B16F10 **C.** and 4T1 **D.** cells were cotransfected with pCLNCX-6× HRELuc and pTCYLacZ plasmids. At 6 h post-transfection, their luciferase activities were determined and normalized with β-gal activity. Multiplicity of infection (MOI). The data shown are the mean ± SD (*n* = 4). * *p* < 0.05; ** *p* < 0.01.

**Figure 4:**
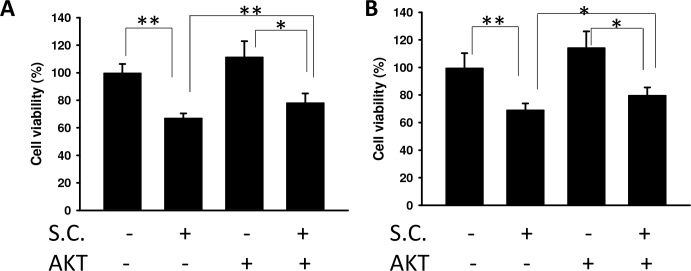
The effect of *Salmonella* on AKT phosphorylation and endothelial cell proliferation The B16F10 **A.** and 4T1 **B.** cells transfected with control or constitutively active AKT plasmids were treated with *Salmonella* (MOI=10). The conditioned medium of those cells reduced the proliferation of endothelial cells. The HEMC-1 cells treated with conditioned medium from *Salmonella*-treated tumor cells were examined using the proliferation activity. Cell viability was assessed by the WST-1 assay. The data shown are the mean ± SD (*n* = 4). Multiplicity of infection (MOI). * *p* < 0.05; ** *p* < 0.01.

We found that *Salmonella* decreased VEGF expression in tumor cells by reducing AKT phosphorylation. The AKT/mTOR/p70S6K signaling pathway was reversed by transfecting a plasmid bearing a constitutively active form of AKT. The suppressive effect of *Salmonella* on the AKT/mTOR/p70S6K signaling pathway was relieved by transfecting a constitutively active form of AKT in B16F10 (Figure 3A) and 4T1 (Figure 3B) cells. Transfection of a plasmid encoding constitutively active AKT slightly increased the expression of HIF-1α and VEGF by *Salmonella* treatment in comparison with the control group. The HIF-1 transcriptional activity was also rescued after *Salmonella* treatment by transfecting a plasmid bearing a constitutively active form of AKT (Figure 3C and 3D). There was a decrease in the proliferation of endothelial cells treated with conditioned medium from tumor cells infected with *Salmonella*. The phenomenon was reversed after transfecting a plasmid encoding constitutively active AKT in two tumor cell lines (Figure 4). Our results suggest that downregulation of AKT is required for the reduction of VEGF expression in tumor cells treated with *Salmonella*.

### *Salmonella* inhibited tumor growth *in vivo*

**Figure 5:**
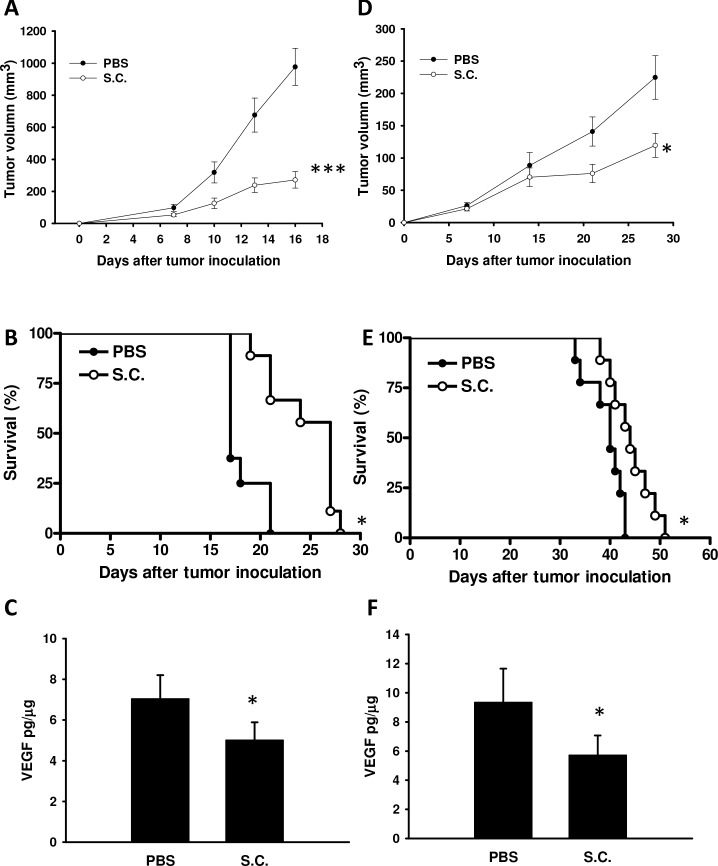
*Salmonella* (S.C.) retarded tumor growth and enhanced the survival of tumor-bearing mice Groups of mice that had been inoculated subcutaneously with B16F10 and 4T1 at day 0 were treated i.p. with *Salmonella* (10^6^ CFU) at day 8. Vehicle control mice were injected with PBS. The B16F10 **A.** and 4T1 **D.** tumor volumes were measured every 3 or 7 days after injection of *Salmonella* (*n* = 8-9, the data are the mean ± SEM. * *p* < 0.05; ***p < 0.001). Kaplan-Meier survival curves of mice bearing **B.** B16F10 melanomas and **E.** 4T1 tumors exposed to various treatments are shown. The effect of *Salmonella t*reatment on VEGF levels in tumor tissue homogenates. Groups of mice that had been inoculated subcutaneously with B16F10 and 4T1 at day 0 were treated i.p. with *Salmonella* (10^6^ CFU) at day 8. Vehicle control mice were injected with PBS. The VEGF levels in B16F10 **C.** and 4T1 (F) tumors were measured by ELISA at day 15 after injection of *Salmonella* (*n* = 4, data are mean ± SD). * *p* < 0.05; *** *p* < 0.001.

**Figure 6:**
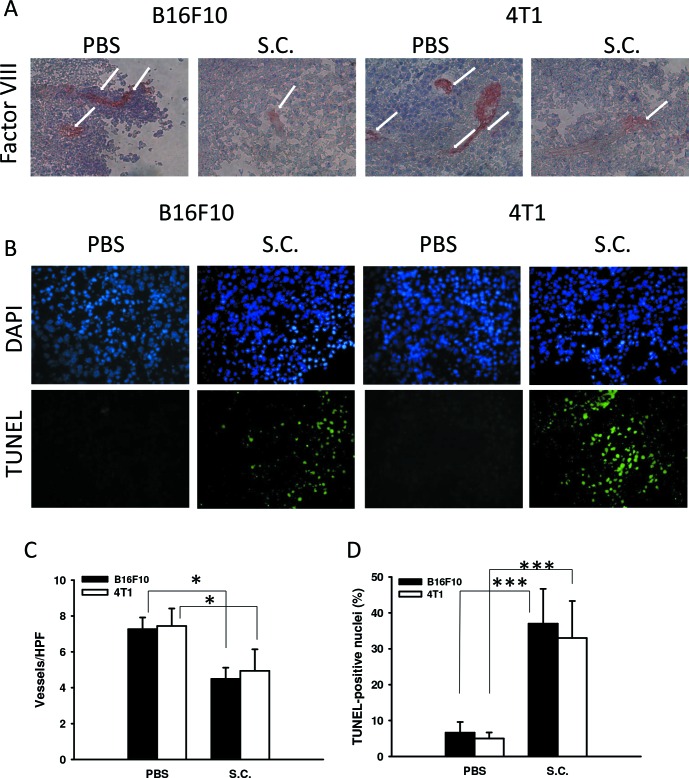
*Salmonella* reduced vessel density and enhanced tumor cell apoptosis **A.** Tumors were excised at day 14, snap frozen and immunostained with rabbit antibody against factor VIII-related antigen (× 400). **B.** Tumors were excised at day 14, and a TUNEL assay was used to detect apoptotic cells (× 400). **C.** Intratumoral microvessel density was determined by averaging the number of vessels in three areas of highest vessel density at × 400 magnification in each section (*n* = 3, the data are the mean ± SD. *p < 0.05). **D.** TUNEL-positive cells were counted from three fields of high-density positive cells in each section to determine the percentage of apoptotic cells (*n* = 3, the data arethe mean ± SD. ***p < 0.001).

The antitumor effects of *Salmonella* were evaluated in terms of tumor growth in mice bearing B16F10 or 4T1 tumors. As shown in Figure 5A and 5D, tumor growth was significantly retarded in mice treated with *Salmonella* in comparison with PBS-treated control mice. Figure 5B and 5E demonstrate that survival of the mice injected with *Salmonella* was significantly prolonged compared with mice injected with PBS. To investigate anti-angiogenesis *in vivo* after *Salmonella* treatment, mice bearing tumors were injected with *Salmonella*, and the levels of VEGF, the microvessel density, and the number of apoptotic cells in the tumors were determined by enzyme-linked immunosorbent assay (ELISA), immunohistochemistry, and terminal dUTP nick-end labeling (TUNEL), respectively (Figure 5C and 5F, and Figure 6). *Salmonella* reduced the levels of VEGF in tumor tissue homogenates (Figure 5C and 5F). Microvessel density within tumors from tumor-bearing mice was analyzed 7 days after *Salmonella* treatment by immunohistochemistry. The results of immunohistochemical staining are shown in Figure 6A. Tumors from *Salmonell*a-treated mice appeared much less vascularized than their control counterparts (Figure 6C). TUNEL assays showed an increase in the amount of cells undergoing apoptosis in the *Salmonella*-treated tumors compared with PBS-treated tumors (Figure 6B). There was a 5.576.62-fold increase in the number of apoptotic cells induced by *Salmonella* compared with PBS-treated tumors (Figure 6D). Taken together, these results indicate that systemic delivery of *Salmonella* can delay tumor growth by reducing angiogenesis in tumors and enhance tumor cell death.

## DISCUSSION

*Salmonella* may inhibit the proliferation and migration of tumor cells. Indeed, it has been demonstrated that VEGF directly stimulates the growth of tumor cells [20, 21]. *Salmonella* inhibited the proliferation of endothelial cells and reduced the production of paracrine factors such as VEGF, thus suppressing the proliferation of tumor cells. *Salmonella* suppressed the expression of VEGF in tumor and angiogenic signaling cascades induced by HIF-1α. The anti-angiogenic effect of *Salmonella* may be similar to anti-VEGF antibody treatment, pruning immature vessels in tumor sites. The vessel normalization and restoration of pressure gradients induced by VEGF blockade may explain the increased replication of *Salmonella* in tumor sites and enhance the antitumor activities of *Salmonella* [15]. Anti-angiogenic agents may contribute to improving the hypoxic condition of tumor sites by vascular normalization [6, 22]. Hypoxia, a hallmark of many solid tumors, was reduced by angiogenic inhibitors [23]. Herein, we showed that *Salmonella* had the ability to reduce HIF-1α expression and may improve the hypoxic condition in the tumor microenvironment as well as increase the effects of radiation or chemotherapy. Indeed, the capability of *Salmonella* to disperse within tumors, and hence to reduce tumor growth, was augmented when combined with chemotherapy [15]. Several activities of *Salmonella* contribute to its antitumor effects. *Salmonella* has the ability to stimulate host immunity and increase tumor suppressor gene expression such as *connexin 43* (*Cx43*) [24–26]. Regulation of *Cx43* activity by *Salmonella* probably contributes to the anti-angiogenic activity [17]. Therefore, it is plausible that the antitumor effect of *Salmonella* may be attributed not only to its effects on tumor endothelial cells but also to its ability to enhance inflammation within tumor sites [24]. In addition, Western blot analysis showed that the expression levels of phospho-AKT (P-AKT), phospho-mTOR (P-mTOR), and phospho-p70s6K(P-p70s6K) in tumor cells were decreased after *Salmonella* infection. Activation of AKT is one of the major mechanisms of tumorigenesis, and blocking this signaling pathway could have therapeutic implications for tumors. Previous studies demonstrated that the activation of AKT is associated with intrinsic radioresistance, tumor cell proliferation, and angiogenesis *in vivo* [27]. AKT can affect proliferation signaling and induce anti-apoptotic effects in tumors. Transgenic mice expressing active AKT develop thymoma and mammary tumors [27, 28]. Our results indicate that *Salmonella* inhibits HIF-1α expression *via* downregulation of the AKT/mTOR pathway. Herein, we demonstrate that *Salmonella*-mediated AKT/mTOR downregulation is an important modulator of HIF-1α expression and plays a crucial role in antitumor therapy.

*Salmonella* had demonstrated immunopotentiating properties [29] and anti-angiogenic activity [30]. The induction of tumor necrosis factor-α in host cells acts as a vascular disrupting agent after *Salmonella* treatment [31]. Meanwhile, *Salmonella* inhibits the expression of VEGF in tumor-associated macrophages [32]. However, the successful induction of antitumor effects in subcutaneous tumors may not necessarily indicate efficacy against orthotopic tumors. Therefore, orthotopic tumor models are preferred for analyzing the tumoricidal effect of *Salmonella* [33, 34]. Our previous results revealed that *Salmonella* accumulated in not only subcutaneous but also orthotopic tumors after systemic administration [35]. *Salmonella* may be beneficial to cancer treatment in the future.

## MATERIALS AND METHODS

### Bacteria, cell lines, plasmids and mice

A vaccine strain of *Salmonella enterica* serovar *choleraesuis* (*S.choleraesuis*) (ATCC 15480) was obtained from the Bioresources Collection and Research Center (Hsinchu, Taiwan). This rough variant of *S. choleraesuis* (S.C.) was designated vaccine 51 [26]. Murine melanoma B16F10 cells [36] and murine breast cancer 4T1 cells [37] were cultured in Dulbecco's modified Eagle's medium (DMEM) supplemented with 50 μg/ml gentamicin, 2 mM L-glutamine, and 10% heat-inactivated fetal bovine serum (FBS) at 37°C in 5% CO_2_. Human HMEC-1 microvascular endothelial cells [14, 17] were cultured in EGM endothelial growth medium (Cambrex, East Rutherford, NJ, USA). Constitutively active AKT plasmid was kindly provided by Dr. Chiau-Yuang Tsai (Department of Molecular Immunology, Osaka University) [38]. The 24-bp HRE (5′-CAC ACG TGG GTT CCC GCA CGT CCG-3′) of the human lactic dehydrogenase A gene was obtained by polymerase chain reaction, and 6 copies of this fragment were individually tandemly ligated into the 5′ region of the CMV minimal (CMVmini) promoter derived from the pTRE vector (Clontech, Palo Alto, CA) at the *Stu*I/*Eco*RI sites. To construct luciferase reporter plasmids, six copies of HRE ligated to the CMV minimal promoter (6×HRE/CMVmini) were excised from the pTRE-based plasmids by digestion with *Kpn*I and *Hin*dIII and subcloned into pGL3 (Promega, Madison, WI) at the *Kpn*I/*Hin*dIII sites. To construct the lentiviral vector, the fragment of 6×HRE/CMVmini-Luc was excised from pGL3 by digestion with *Kpn*I/*Bgl*II and subcloned into pMECA. The fragment of 6×HRE/CMVmini-Luc was released from the pMECA-based plasmid by digestion with *Cla*I/ *Swa*I sites and cloned into pWPXL [16]. Six-to-eight-week-old female BABL/c and C57BL/6 mice were obtained from the National Laboratory Animal Center of Taiwan. The animals were maintained in a specialized pathogen-free animal care facility in isothermal conditions with regular photoperiods. The experimental protocol adhered to the rules of the Animal Protection Act of Taiwan and was approved by the Laboratory Animal Care and Use Committee of the China Medical University (permit number: 104-24-N).

### Endothelial cell viability assay

HMEC-1 cells (2 × 10^3^/well) were cultured in 96-well plates. Tumor cells (10^5^/well) that had been cultured in 6-well plates overnight were infected with various MOIs of *Salmonella* or mock-infected with antibiotic-free culture medium for 4 h. The medium was then removed, and cells were washed and replenished with fresh medium supplemented with 2% FBS and 50 μg/ml gentamicin. After 48 h, the conditioned medium was collected, filtered through a 0.22-μm filter, and analyzed for its ability to inhibit endothelial cell proliferation. The culture HMEC-1 medium was then removed and replaced with conditioned medium. After 48 h, cell proliferation was assessed by the colorimetric WST-1 assay (Dojindo Labs, Tokyo, Japan) according to the manufacturer's instructions [38].

### Analysis of hypoxia-inducible transcriptional activities

Various cells grown in 24-well plates were cotransfected with luciferase reporter plasmids driven by HRE promoters (0.66 μg) and pTCYLacZ (0.34 μg), a β-galactosidase (β-gal) expression plasmid driven by the β-actin promoter, using Lipofectamine 2000. The culture medium was then removed and replaced with conditioned medium. At post-transfection, cell lysates were harvested 16 h later. The cell lysates were assessed for their luciferase activities, as determined by a dual-light luciferase and β-gal reporter gene assay system (Applied Biosystems, Foster City, CA, USA) using a luminometer (Minilumate LB9506, Bad Wildbad, Germany). Relative luciferase activity was measured as luciferase activity divided by β-gal activity to normalize transfection efficiency per microgram protein. The protein content in each sample was determined by the bicinchoninic acid (BCA) protein assay (Pierce Biotechnology, Rockford, IL). The cells with high levels of luciferase expression were transfected with control or constitutively active AKT plasmids. The luciferase activity was assessed by a luminometer as previously described.

### Animal studies

Groups of mice were subcutaneously (s.c.) inoculated with 106 tumor cells. When the tumors had grown to diameters between 50 and 100 mm^3^, the mice were intraperitoneally (i.p.) injected with 2 × 10^6^ colony-forming units (cfu) of *Salmonella*. Palpable tumors were measured every 3 days or 7 days along two perpendicular axes using a tissue caliper, and the tumor volumes were calculated as follows: (length of tumor) × (width of the tumor) ^2^ × 0.45. All mice were monitored for tumor growth and survival as previously described [14].

### Immunoblot analysis

The protein content in each sample was determined using a BCA protein assay (Pierce Biotechnology). Proteins were fractionated by SDS-PAGE, transferred onto Hybond enhanced chemiluminescence nitrocellulose membranes (Amersham, Little Chalfont), and probed with antibodies against HIF-1α (Novus Biologicals, Littleton, CO), VEGF (Novus Biologicals), the mammalian target of rapamycin (mTOR) (Cell Signaling, Danvers, MA, USA), phospho-mTOR (Cell Signaling), AKT (Santa Cruz Biotechnology, Inc. Santa Cruz, CA, USA), phospho-AKT (Santa Cruz Biotechnology, Inc.), p70 S6 kinase (p70S6K) (Cell Signaling), phospho-p70S6K (Cell Signaling), or monoclonal antibodies against β-actin (AC-15, Sigma Aldrich). Horseradish peroxidase-conjugated goat anti-mouse IgG or anti-rabbit IgG (Jackson, West Grove, PA, USA) was used as the secondary antibody, and protein-antibody complexes were visualized by an enhanced chemiluminescence system (Amersham). The signals were quantified with ImageJ software (rsbweb.nih.gov/ij/) [39].

### ELISA, immunohistochemistry and TUNEL

The levels of mouse VEGF in tumor tissue homogenates were determined by ELISA [17]. To analyze microvessel density in the tumor sites, the whole tumors were excised and snap frozen at day 14. Frozen tumor sections were prepared according to the aforementioned procedure, and incubated with rabbit anti-factor VIII-related antigen (DAKO, Carpinteria, CA). After sequential incubation with appropriate peroxidase-labeled secondary antibody and aminoethyl carbazole (AEC) as the substrate chromogen, tumor sections were counterstained with hematoxylin. Areas containing the highest number of capillaries were identified by scanning the tumor sections at × 100 magnification. After the fields of high microvessel density (neovascular “hot spots”) were determined, individual vessels were counted in × 400 magnifications. Microvessel density was determined by averaging the number of microvessels in the three areas of highest vessel density at × 400 magnification in each section. The orientation of the sections was random [14, 17]. A TUNEL assay was used to detect cell death in tumors and was performed according to the manufacturer's instructions (Promega, Madison, WI). We used three high-power (× 400) fields with approximately 200-300 cells that showed the highest density of positive-stained cells per field to determine the average percentage of apoptotic (TUNEL positive) cells in each section [35].

### Statistical analysis

The unpaired, two-tailed Student's t test was used to determine differences between groups for comparison with the control group. A survival analysis was performed using the Kaplan-Meier survival curve and log-rank test. A *p* value less than 0.05 was considered to be statistically significant.
